# Volcanic passive margins: another way to break up continents

**DOI:** 10.1038/srep14828

**Published:** 2015-10-07

**Authors:** L. Geoffroy, E. B. Burov, P. Werner

**Affiliations:** 1 Université de Bretagne Occidentale, Brest, 29238 Brest; 2 CNRS, UMR 6538, Laboratoire Domaines Océaniques, 29280 Plouzané, France; 3 Sorbonne Universités, UPMC Université Paris VI, 75005 Paris, France; 4 CNRS, UMR 7193, Institut des Sciences de la Terre Paris (iSTeP), 75005 Paris, France; 5 TOTAL, Exploration-Production, 92078 Paris la Défense Cedex, France

## Abstract

Two major types of passive margins are recognized, i.e. volcanic and non-volcanic, without proposing distinctive mechanisms for their formation. Volcanic passive margins are associated with the extrusion and intrusion of large volumes of magma, predominantly mafic, and represent distinctive features of Larges Igneous Provinces, in which regional fissural volcanism predates localized syn-magmatic break-up of the lithosphere. In contrast with non-volcanic margins, continentward-dipping detachment faults accommodate crustal necking at both conjugate volcanic margins. These faults root on a two-layer deformed ductile crust that appears to be partly of igneous nature. This lower crust is exhumed up to the bottom of the syn-extension extrusives at the outer parts of the margin. Our numerical modelling suggests that strengthening of deep continental crust during early magmatic stages provokes a divergent flow of the ductile lithosphere away from a central continental block, which becomes thinner with time due to the flow-induced mechanical erosion acting at its base. Crustal-scale faults dipping continentward are rooted over this flowing material, thus isolating micro-continents within the future oceanic domain. Pure-shear type deformation affects the bulk lithosphere at VPMs until continental breakup, and the geometry of the margin is closely related to the dynamics of an active and melting mantle.

In general terms, if we consider frictional and non-linear ductile behavior of rocks and extension rates of the order of a few cm/yr, the extension of “normal” continental lithosphere requires high levels of differential stresses^1^,^2^,^3^. However, passive margins record events that end with continental break-up, when the integrated strength of rifted lithosphere drops to zero^1^,^3^. The role of magma intrusion in favoring and focusing extension may be important as the lithosphere can be both thermally weakened by hypothetical lithospheric-scale dykes^4^ or compositionally strengthened in lower crustal section by cooled mafic intrusions^5^.

The distinction between between the volcanic (VPMs) and non-volcanic (in this case, sedimentary) passive margins (SPMs) is basically drawn on the basis of timing and degree of mantle melting in relation to lithosphere extension, break-up and plate separation^6^,^7^,^8^,^9^.

SPMs show no record of significant mantle melting in their upper crustal section immediately before and throughout lithosphere extension^10^. In such settings, sub-crustal mantle can be exhumed and serpentinized in association with extreme crustal stretching and thinning (Fig. 1)^5^,^6^,^7^,^8^,^9^,^10^,^11^,^12^,^13^. Lithosphere necking is accommodated by an early system of upward-concave conjugate detachment faults dipping “oceanward”^5^,^13^ (Fig. 1a). A major trans-lithospheric detachment developing seaward would finally exhume the upper lithospheric mantle through a rolling-hinge deformation of the footwall^5^,^12^ (Fig. 1b). Asthenospheric mantle melting and generation of MORB-type magmas would finally occur through impingement of the main detachment fault^13^,^14^. An alternative model invoking the exhumation of a mechanically weak lower crust has also been proposed for SPMs^15^.

VPMs characterize continental break-up in Large Igneous Provinces (LIPs)^6^,^7^,^8^,^9^. As exemplified in the Afar area, significant lithosphere extension does not appear to be a prerequisite for initial mantle melting and consecutive syn-magmatic break-up^8^,^9^. Early melt produces volcanic traps that cover large areas including continental cratons and/or craton edge (up to 10^7^ km^2^ or more)^6^,^7^. During this early stage, massive crustal dilatation occurs through dyking in the upper crust^16^ and magma underplating at the Moho^17^. Lithosphere extension leading to break-up is coeval with focusing of mantle melting, giving rise to VPMs^6^,^7^,^8^. The proximal and upper crustal parts of VPMs are exposed along the coast of central W- and E-Greenland, giving access to the extrusive section and the underlying sheeted dyke complexes intruded through the continental basement^18^,^19^.

In the present study we investigate the mechanisms of stretching and thinning of the continental lithosphere in magma-rich settings, based on new observational data and the results of physically consistent thermo-mechanical numerical modelling. We use a new set of long-offset commercial seismic reflection data from VPMs worldwide to study their deep structure down to ~40 km. For that we have selected two ION Geophysical dip-lines across the Pelotas volcanic margin in the southern Atlantic (Fig. 2a). The Pelotas and Namibia conjugate VPMs formed within the Gondwana-related Mantiqueira Province after the onset of eruption of the Parana-Etendeka volcanic traps during the Hauterivian^20^,^21^,^22^,^23^. The time-span of syn-magmatic continental lithosphere stretching/thinning is bracketed between ~130 Ma (end of traps emplacement) and ~115 Ma^21^. The magma budget of the Pelotas margin varies along-strike depending on its location with respect to the Rio Grande Rise (Fig. 2a,b)^21^: Line PS1-0090 (Fig. 2b) lies across a particularly magma-rich segment compared to Line PS1-0040 (see Fig. 1 in Extended Data). Although the ION Geophysical PelotasSPAN data set has recently been analysed^21^, we present here a different profile (PS1-0040; Fig. 1 in Extended Data) along with different interpretations. We also use seismic refraction and potential data from the conjugate margin of Namibia south of Walvis Rise^22^,^23^, which are further constrained by a set of recent seismic reflection profiles (down to ~8–9 sec two-way travel time) as shown in Extended Data Fig. 3.

From top to bottom of the most proximal (i.e. continental) part of the Pelotas margin, we distinguish four crustal entities (Fig. 2b; Figs 1 and 2 in Extended Data): (1) an extrusive section (forming typical wedges of Seaward-Dipping Reflectors Sequences^24^, abbreviated here as SDRs) up to 20 km in total thickness, with distinct units bounded by continentward-dipping syn-extrusive growth detachment faults (CDFs)^19^,^25^,^26^,^27^; (2) an intruded upper crust (UC) underlying the SDRs^18^,^19^; (3) a middle-lower crust (LC1); and, (4), a ~4 km-thick magmatic crust (LC2) characterized by stacked reflections. In agreement with onshore observations, SDR wedges develop seaward with time^18^,^19^, associated with progressive inactivation and migration of the SDR-bounding faults^19^. Individual CDFs exhibit cumulated throws of more than 5–6 km with, in places, allochthons of upper-crustal blocks (UC) at their footwalls. The deepest and oldest CDFs generally have the lowest dip, being rotated clockwise by the flexure of the more recent upper levels^19^. CDFs are frequently picked out by strong reflections suggesting they are magma-injected^24^,^25^. It is noteworthy that these faults flatten out over the top of the middle/lower crustal level LC1 or are seen to be rooted within it. The LC1 layer is highlighted by a number of strong reflections of probable intrusive origin locally displaying fold-like structures (Fd in Fig. 2b; see Figs 1 and 2 in Extended Data), suggesting that bulk solid-state flow postdates the main intrusive stage. On line PS1-0090, the ductile LC1 is clearly structurally disconnected from LC2, which we interpret, as other authors^28^, as representing the stacking of mafic to ultramafic sill-like intrusions at the crust-mantle transition. The LC2 layer loses its coherent layered pattern seaward, becoming undistinguishable from the disrupted LC1 (Fig. 2b). The distinct behaviour of LC2 in relation to LC1 in the innermost part of the Pelotas margin may stem from compositional differences since the corresponding layer in Namibia yields the highest seismic velocities^22^. The LC2 layer would appear to be almost entirely mafic (sheeted sill complex) and thus more rigid than the (relatively) less intensively intruded LC1. The magmatic accretion of both LC1 and LC2 probably began during the “trap stage”^17^, and continued during the stretching and thinning of the lithosphere.

The crustal profiles observed on lines PS1-0040 and PS1-0090 indicate a strong and narrow syn-magmatic necking of the upper crust, with a deep and relatively flat-lying Moho at the continent-ocean transition. The Moho has gentler dips and is significantly deeper (33 +/− 1 km vs. 25 +/− 5 km) in the proximal part of the magma-rich VPM (Fig. 2b; Fig. 1 in Extended Data). In all our studied cases of VPMs, the transitional crust is clearly thickened in the necking area by magma accretion, due to the intrusion of sills in the lower crust and extrusion of lavas at the surface (Figs 1 and 2 in Extended Data). Therefore, crustal extension is not isovolumic at VPMs and pre-extension geometries can hardly be restored. Because the sills in LC1 seem disrupted by ductile flow in most of our studied profiles, we infer early magma injection continuing throughout the thinning/stretching process to account for the syn-tectonic formation of SDRs. The necked upper crust is also strongly intruded (Fig. 2b), in this case by dykes, which are responsible for significant horizontal dilatation^18^,^19^. In agreement with other authors^20^,^21^,^22^,^23^, we find no evidence of mantle exhumation based on the attitude of the Moho at the continent-ocean transition (Fig. 2b). The adjoining oceanic crust (thickness ~7 + /− 1 km) has a lower section characterized by magma injected in faults dipping towards the oceanic spreading ridge (Fig. 1 in Extended Data).

The conjugate Namibia margin^20–23^ shows similar features in the upper crustal section, notably the SDR wedges bounded by CDFs (Fig. 3a,b in Extended Data). Our reconstruction at the time of break-up (Fig. 2c) emphasizes the importance of magma accretion at these margins, and highlights the extreme upper-crustal thinning and the apparent spreading of the intruded lower crust beneath relics of the brittle crust.

From our deep seismic data set, the continent-ward dip of large syn-extrusive detachment faults connected to a flowing lower crust (LC1), thus appears to be a characteristic feature of conjugate VPMs during necking, which is a strikingly opposite pattern compared with non-volcanic margins (Fig. 1). To understand the causes of this phenomenon, we used a fully coupled thermo-mechanical 2D code Flamar^29^,^30^ (see Methods) to model extension of continental lithosphere in a mantle-melting context. In the models, the lithosphere rheology accounts for cold and highly viscous low-Si0_2_ magmatic rocks emplaced in the lower crust at early LIP stages^17^ and also newly-formed magma intruded and extruded during lithosphere stretching and thinning. We assume a high-viscosity layer in the lowermost crust before applying a symmetrical 1.5 cm/yr extensional displacement at each side of the model (Fig. 3a). New magma is produced continuously as a function of lithosphere thickness and mantle potential temperature^31^ (see Methods). The strength of the mantle lithosphere, which is apparently weak at VPMs^19^, is limited by Pierls-type behaviour^32^ and by imposing a slight excess in temperature (50 °C) (Fig. 3a; see Methods).

Both weak lithospheric mantle and initially strong lower crust, are *sine qua non* preconditions for the development of early conjugate CDFs. The footwall of the two opposite CDFs forms a central rigid continental block (C-Block in Fig. 3b,c), which becomes isolated in less than 1 Ma after the onset of extension (Extended Data Fig. 4). The development of crustal-scale conjugate detachments dipping outward with respect to the C-block is primarily due to the thermally-driven weakening of the mantle lithosphere, which partly flows outward and upward along the bottom of the C-block hardened by the initial “underplating” (Fig. 3b,c; Fig. 4 in Extended Data). This outward mantle flow is partly decoupled from the continental crust. This flow mechanically erodes laterally outward the lowermost parts of the C-block which, from ~2 Ma, becomes restricted to the rigid upper crust (Fig. 3c; Fig. 3 in Extended Data). Although we did not model anatectic processes erosion of the C-Block is probably enhanced by the partial melting of its lower and middle crust^33^. This, lateral flow creates a bulge of mixed rigid (mafic) and ductile (felsic) crust at the edges of the C-block (Fig. 3b,c). This bulge then localizes the detachments controlling the formation of SDRs. The magma, ascending from the asthenospheric mantle, also flows laterally outward and upward along the bottom of the C-block and is trapped within the detachment faults (Fig. 3b,c). From the outset, the magma-assisted concave-upward CDFs are purely crustal structures which develop in response to gravity collapse of the thick and denser non-deformed continental crust in relation to the thin and buoyant central part of the model (C-block) (Figs 3c and 4). While our model does not aim to reproduce all the small-scale characteristics of conjugate VPMs, it provides new insights on the origin of the inner SDRs which are coeval with crustal necking at VPMs. The seaward blocks observed at VPMs (e.g. Figs 1 and 2b in Extended Data), as well as the outer-SDRs, probably result from the progressive shredding of the C-Block with time (Fig. 4). Thus, more or less dissected micro-continents would be individualized within the oceanic domain, being restricted to the upper crust (Fig. 4). Although microcontinents may have different origins^34^, there is increasing evidence of such buoyant continental blocks being “lost” within oceans when continental breakup occurs in hot-mantle environments^35^ such as notably in the South Atlantic, offshore from the studied margins^21^,^36^.

Figure 5 takes into account the onshore, offshore and modelling data to summarize the main stages in the evolution and final structure of VPMs, as compared to SPMs (Fig. 1). Extreme crustal thinning and stretching leads to break-up (i.e. oceanization) in the case of both SPMs (Fig. 1b) and VPMs (Fig. 5c), but lithospheric pure shear (associated with continent-ward dipping shear-zones at both margins) dominate over simple shear at VPMs. Cold lithospheric mantle may be exhumed (and serpentinized) in SPMs, whereas the geodynamic evolution of VPMs involves a hot active and melting mix of sub-Moho and asthenospheric mantle, which never appears to reach the ocean floor (Fig. 5b,c).

## Methods

### Numerical model

**Figure 3:**
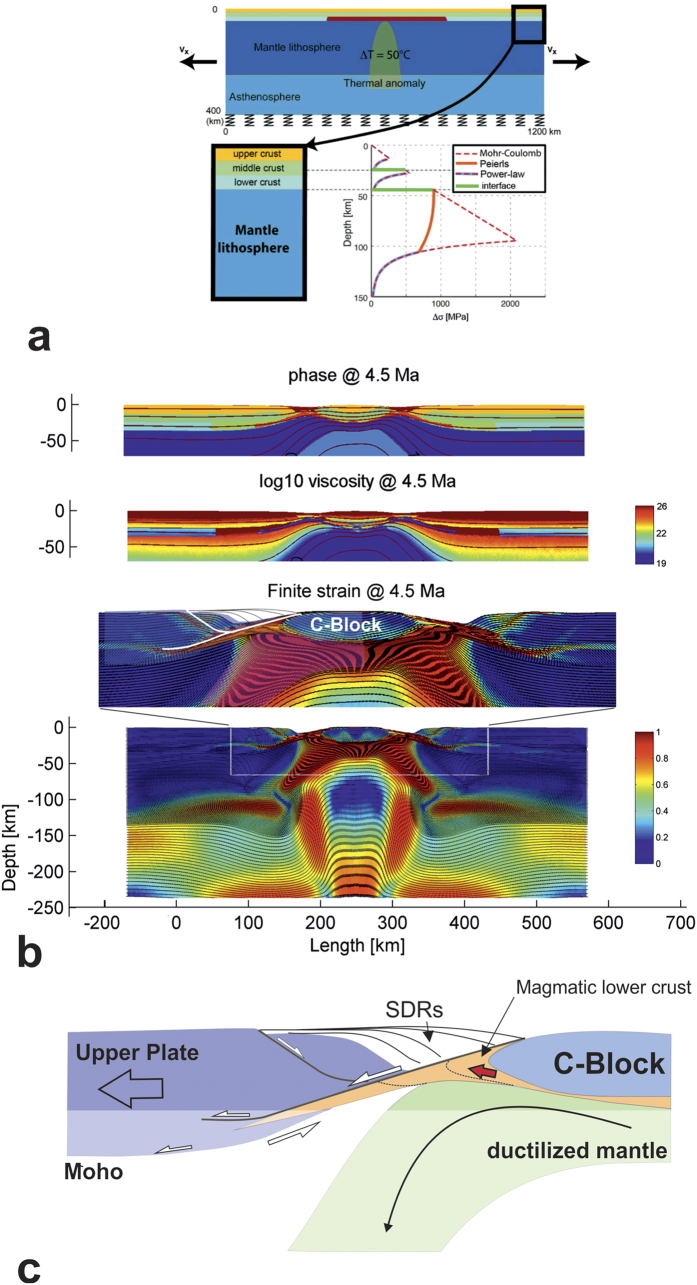
(**a**) Numerical model setup (see Methods). Red colour in the upper panel corresponds to magmatic underplating (viscosity is ~10^24^ to 10^25^ Pa.s and 10^21^ Pa.s for the ambient lower crust). An initial, negligibly small thermal anomaly (50 °C) is used to initialize rifting in the middle of the model, which dissipates soon afterwards. The bottom right panel shows a typical viscous-elastic-plastic lithospheric strength profile used in the numerical experiments. (**b**) 4.5 Ma after imposing a 1.5 cm/yr displacement on each side of the model. From top to bottom are shown: the material phase field, the effective viscosity field (in Pa.s) and the finite strain field. Note that the rheology used is viscous-elastic-plastic, so effective viscosity is defined as the ratio of deviatoric stress to strain rate, for illustrative purposes only. (**c**) Tectonic/dynamic sketch related to the left part of the enlarged finite strain panel in (**b**). Authors: E.B (modelling) and L.G. (interpretation). Images created from modelling results using Adobe Photoshop CS6 (results) and CorelDraw11 (interpretation).

We use a numerical thermo-mechanical modelling approach based on the numerical code Flamar v12^29^,^30^ to assess the response of the lithosphere under various thermo-rheological conditions. This thermo-mechanical code handles free surface boundary conditions which allow the modelling of the topography and basement evolution, as well as the large strains and visco(ductile)-elastic-plastic(brittle) rheologies characteristic of different lithospheric and mantle units (see Supplementary Information). These conditions include Mohr-Coulomb failure for brittle deformation (faulting), pressure-temperature strain-rate dependent ductile flow for viscous deformation, thermo-dynamic phase transitions, internal heat sources and elastic compressibility. The code incorporates particle-in-cell remeshing and tracking of trajectories of particles. For the numerical simulations, we use the model setup shown in Fig. 3a. The multilayered visco-elasto-plastic continental lithosphere is composed of a 40 km-thick crust, with a 20-km-thick upper crustal layer exhibiting a dry granite rheology underlain by a 20 km-thick dry diabase lower crust. The total thickness of the lithosphere is 150 km. The densities are updated dynamically as a function of pressure and temperature using thermo-dynamic free-energy minimization. The base of the model is at 400 km. The initial thermal gradient in the lithosphere is computed as a function of its age using a half-space cooling model that accounts for radiogenic heat sources. The initial temperature at the base of the lithosphere is 1330 °C and the initial linear thermal gradient in the underlying mantle is such that it yields a temperature of 1700 °C at 650 km depth (the bottom of the upper mantle). The temperature at factual bottom of the model (400 km depth) is derived from this condition. Zero thermal out-flux is used as a lateral boundary condition. The mechanical boundary conditions are as follows: free upper surface, reflecting boundary conditions or horizontal velocities at the lateral borders, and a hydrostatically compensated bottom.

The numerical code Flamar is based on the FLAC^37^ and Parovoz algorithm^38^, as described in many previous studies^30^,^39^,^40^,^41^,^42^. For this reason, we limit the description of the code to the essential features: the ability to handle (1) large strains and multiple visco-elastic-plastic rheologies (EVP) including Mohr-Coulomb failure (faulting) and non-linear pressure-temperature and strain-rate dependent creep; (2) strain localization; (3) thermo-dynamic phase transitions; (4) internal heat sources; (5) free surface boundary conditions and surface processes.

### Basic equations

Flamar has a mixed finite-difference/finite element numerical scheme, with a 2D Cartesian coordinate frame (*x*_*i*,_ i = 1, 2) and 2D plane strain formulation. The Lagrangian mesh is composed of quadrilateral elements subdivided into 2 couples of triangular sub-elements with tri-linear shape functions. Flamar uses a large strain fully explicit time-marching scheme. It locally solves full Newtonian equations of motion in a continuum mechanics approximation:


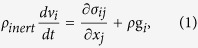


where σ is full stress, i.e. 
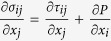
, *P* is pressure, τ is deviatoric stress, *ρ* is density, *ρ*_*inert*_ corresponds to the inertial density^37^, i.e. *ρ*_*inert*_ = *ρ* in the dynamic mode or 
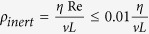
 in quasi-static mode, where *v* is velocity, *L* is the characteristic flow length, *η* and is dynamic viscosity, Re is the “numerical” Reynolds number that must be smaller than 0.01 in a quasi-static regime. The equation (1) is coupled with constitutive equations:


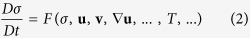


and using equations of heat transfer, with a heat advection term included in the Lagrangian derivative *DT/D*t, as follows:


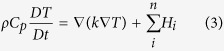



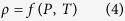


Here, **u**, ***g***, *k* are the respective terms for displacement (***v*** = 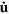), acceleration due to body forces and thermal conductivity. The terms *t*, *Cp*, *T*, *H*_*i*_ designate time, specific heat, temperature and internal heat production per unit volume, respectively. The expression *ρ* = *f(P*, *T)* refers to the formulation, in which phase changes are taken into account and density is computed by a thermodynamic module^43^ that evaluates the equilibrium density of constituent mineralogical phases for given *P* and *T* as well as the contribution of latent heat *H*_*l*_ to the term 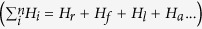, which also accounts for radiogenic heat, *H*_*r*_ , frictional dissipation, *H*_*f*_ and adiabatic heating, *H*_*a*_. The terms *D*σ*/Dt* and *F* are the objective Jaumann stress time derivative and a function, respectively. In the Lagrangian method, incremental displacements are added to the grid coordinates allowing the mesh to move and deform with the material. This enables the solution of large-strain problems while locally applying a small-strain formulation: at each time step, the solution is obtained in local coordinates, which are then updated in a large-strain mode, as in a standard finite element framework.

Solution of Eq. 1 provides velocities at mesh points used for the computation of element strains and heat advection 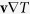. These strains are used in Eq. 2 to calculate the element stresses and equivalent forces used to compute velocities for the next time step. Due to the explicit approach, there are no convergence issues, which commonly arise for implicit methods in cases of non-linear rheologies. The algorithm automatically checks and adopts the internal time step using 0.1–0.5 of Courant’s criterion of stability, which guarantees a stable solution.

### Phase changes

A direct solution for density (Eq. 4), *ρ* = *f(P,T)* is obtained by optimizing Gibbs free energy for a typical mineralogical composition (5 main mineralogical constituents) of the mantle and lithosphere. For this purpose, we couple Flamar with the thermodynamic code PERPLE_X^43^. PERPLE_X minimizes free Gibbs energy *G* for a given chemical composition to calculate an equilibrium mineralogical assemblage for given P-T conditions:


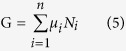


where μ_i_ is the chemical potential and *N*_*i*_ the number of moles for each component *i* forming the assemblage. Given the mineralogical composition, the computation of density is straightforward. Given the mineralogical composition, the computation of density is straightforward. The thermodynamic and solid state physics solutions included in PERPLE_X also yield estimations of the elastic and thermal properties of the materials, which are dynamically integrated, along with the density, into the equations 1, 2, 3, 4 constituting the thermo-mechanical kernel of Flamar.

### Explicit elastic-viscous-plastic rheology

We use a serial Maxwell-type solid in which the total strain increment in each numeric element is defined by the sum of elastic, viscous and brittle strain increments. Flamar explicitly treats all rheological terms. The parameters of elastic-ductile-plastic rheology laws for the crust and mantle are derived from rock mechanics data (Extended Data Table 2).

#### Plastic (brittle) behaviour

The brittle behaviour of rocks is described by Byerlee’s law^44^,^45^, which corresponds to a Mohr-Coulomb material with friction angle *ϕ* = 30° and cohesion |*C*_0_| < 20 MPa:


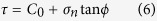


where σ_n_ is normal stress σ_n_ = ⅓σ_I_ + σ_II_^dev^ sin*ϕ*, ⅓σ_I_ = *P* is the effective pressure (negative for compression), σ_II_^dev^ is the second invariant of deviatoric stress, or effective shear stress. The condition of the transition to brittle deformation (function of rupture *f*) can be expressed as: *f* = σ_II_^dev^ + *P* sin*ϕ* − *C*_0_ cos*ϕ* = 0 and ∂*f*/∂*t* = 0. In terms of principal stresses, the equivalent of the yield criterion (8) is given by:


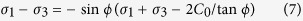


### Elastic behaviour

The elastic behaviour is described by the linear Hooke’s law:


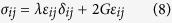


where λ and G are Lamé’s constants. Repeated subscripts imply summation and δ is Kronecker’s operator.

### Viscous (ductile) behavior

Within deep lithosphere and underlying mantle regions, creeping flow is highly dependent on temperature and corresponds to a non-linear non-Newtonian fluid since the effective viscosity also varies as a function of differential stress^3^,^45^,^46^,^47^:


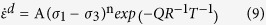


where 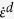 is effective shear strain rate, *A* is a material constant, *n* is the power-law exponent, *Q* = *E*_*a*_ + *PV* is the activation enthalpy, *E*_*a*_ is activation energy, *V* is activation volume, *P* is pressure and *R* is the universal gas constant, *T* is temperature in K, and *σ*_*1*_*, σ*_*3*_ are the principal stresses. In the case of diffusion creep, *A* = *a*^*m*^*A*^*d*^, where *a* is grain size and *m* is an experimental parameter for diffusion creep. The effective viscosity μ_eff_ for this law is defined as:


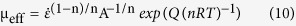


The dominant creep mechanism in the lithosphere is dislocation creep, while diffusion creep plays this role at deeper levels in the sublithospheric mantle. The composite viscosity is calculated according to the generally adopted mixing rule^3^:


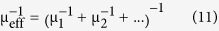


We use an activation volume of 9.5×10^−6^ m^3^ mol^−1 ^ [Ref. 48]. Dislocation creep parameters are provided in the Extended Data Table 2. For diffusion creep, we use an activation energy Ea = 300 KJ mol^−1^, *A*^*d*^ = 1.92 × 10^−10^ MPa^−1^ s^−1^, *a* = 1 and *m* = 1 [Ref. 49]. For non-uniaxial deformation, the law (10) is converted to a triaxial form, using the invariant of strain rate and geometrical proportionality factors^40^:


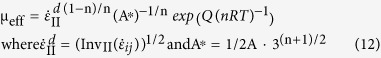


The parameters *A*, *n*, *Q* are experimentally determined material parameters (Extended Data Table 2). Using olivine parameters, we verify that the predicted effective viscosity just below the lithosphere is 10^19^–5 × 10^19^ Pa s, thus matching post-glacial rebound data^50^. Due to temperature dependence of the effective viscosity, the viscosity decreases from 10^25^–10^27^ Pa s to asthenospheric values of 10^19^ Pa s in the depth interval 150–250 km. Within the adiabatic temperature interval in the convective mantle below the lithosphere bottom, the dislocation flow law (Eq. 10) is dominated by nearly Newtonian diffusion creep. In this interval, temperature increases very slowly with depth, while the linear rise in pressure starts to affect viscosity resulting in a slow growth from 10^19^ Pa s in the asthenosphere to a value ranging from 10^20^ Pa s to 10^22^ Pa s at the base of the upper mantle^50^. Due to uncertainties on the viscosity at the base of the upper mantle, we tested several assumptions on creep parameters. Based on these tests, we finally used the assumption of 10^20^ Pa s.

### Surface processes

#### Short-range erosion

A simple law is used to simulate erosion and sedimentation at the scale of a rift basin^30^. The evolution of a landscape results from a combination of weathering processes that prepare solid rock for erosion, and transportation by hillslope and stream processes^51^. Although many factors depending on the lithology and climate may control this evolution, quite simple mathematical models have been proposed and tested successfully for describing the geometrical evolution of the morphology at a small scale^51^. For example, the evolution of scarp-like landforms can be modelled assuming that the rate of downslope transport of debris, *q*, is proportional to the local slope, ∇*h*^51^.


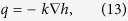


where *k* is the mass diffusivity coefficient, expressed in units of area per time [e.g., m^2^/y]. Assuming conservation of matter along a 2-D section and no tectonic deformation, *h* must obey:


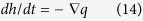


The equations (13 and 14) lead to the linear diffusion equation:


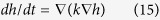


At a larger scale, hillslope and stream processes interact and the sediment transport will then depend non-linearly on the slope and other factors such as the slope gradient, the area drained above a given point and the distance from the water divide, so that simple 2-D linear diffusion does not apply in general. In spite of these limitations, we choose to adopt a linear diffusion law to model erosion. This model does not accurately mimic the spatial distribution of denudation in the mountain range, but it leads to a sediment yield at the mountain front that is roughly proportional to the mean elevation of the basin relative to a given point (a rough approximation to the sediment yield resulting from a change of elevation *h* over a horizontal distance *d* is *k×h/d*), and therefore accounts for the apparent correlation between elevation and denudation rates^30^. We assume a *k* value of 500 m^2^/y, which yields denudation rates of the order of those predicted by previous parametric models^51^.

### Partial melting and magmatic processes

Partial melting and magmatic processes are taken into account following the commonly used simplified parametrization^52^,^53^ of hydrous mantle melting processes for large-scale settings (see full details in ref. 52). According to this approach, melting of hydrated mantle rocks occurs in the P-T region between the wet solidus and dry liquidus of all petrological components. For simplicity, the degree of melting given by the volumetric fraction of melt with temperature *M* is assumed to change linearly with the temperature^52^:


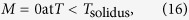



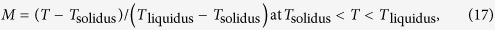



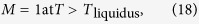


where *T*_solidus_ and *T*_liquidus_ are respectively the wet solidus and dry liquidus temperatures for a given pressure *P*, and rock composition^52^ is:


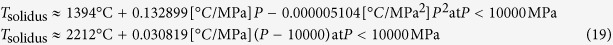


where *P* is in MPa and the square brackets denote the physical units of the corresponding coefficient. The effective viscosity *η* of partially molten rocks is non-linearly dependent on the melt fraction and computed according to the next common approximation (see ref 52 and references therein):


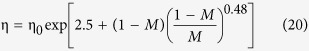


where *η*_0_ ≈10^13^ Pa.s and is the experimentally/empirically defined composition-dependent viscosity parameter for sub-crustal rocks^52^. This yields *η* in the range of 1 × 10^14^ ≤ η ≤ 2 × 10^15^ Pa.s for 0.1 ≤ *M* ≤ 1. The global minimal cut-off viscosity in the experiments was set to 10^15^ Pa.s, so that in practice the magma viscosity was limited from below by this cut-off value. This assumption is valid, since according to the assumed parameterization the viscosity of the host rocks is more than two orders of magnitude higher than that of even slightly molten rock, so the characteristic magma transport time is negligible compared to that of the bulk processes. The density changes due to crystallization of magma and partial melting are taken into account using the general approximation^52^:


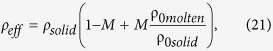


where ρ_eff_, ρ_0solid_, and ρ_0molten_ are respectively the effective density and the reference densities of solid and molten rock (3300 kg/m^3^ and 2700 kg/m^3^), and ρ_solid_ is the density of solid rock at a given *P* and *T* computed by the thermodynamic algorithm PERPLE_X (see section “Phase changes” above).

The effect of latent heating due to equilibrium melting/crystallization is included via the effective heat capacity (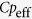) and thermal expansion (α_eff_) of the partially crystallized/molten rocks (0 < *M* < 1)^52^:


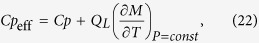



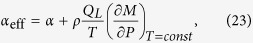


where 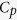 is the heat capacity of the solid rock, *Q*_L_ is the latent melting heat of the rock (400 kJ/kg for mantle rocks^52^), and α is the thermal expansion coefficient of solid rock.

Since the characteristic magma transport time is negligible with respect to that of the bulk deformation processes, the particular effects of melt percolation through the porous matrix can be neglected, so we assume that the melt is extruded in the direction of the pressure difference gradient at a rate (basically >10 m/y) equal to the rate given by the Darcy porous law solution for porous flow in highly permeable rock (permeability of 10^−16^ m^2^). Here, we follow the common simplified approach^52^, which is an acceptable approximation in the absence of reliable data on the dynamic porosity and permeability parameters of the host rocks, and given the relative unimportance of taking into account the fine details of the mechanisms of magma circulation for our problem. In the model, the magma penetration depth through the continental crust is thus controlled by pressure (hence mainly by the density contrast with the host rock; eq. 21) and magma viscosity that exponentially decreases with temperature (eq. 12). That is, when the magma temperature becomes close to that of the surrounding rocks, its viscosity becomes higher than that of the surroundings if the nearby rocks are quartz-rich (e.g. cooled magma sills in the continental crust).

## Additional Information

**How to cite this article**: Geoffroy, L. *et al*. Volcanic passive margins: another way to break up continents. *Sci. Rep*. **5**, 14828; doi: 10.1038/srep14828 (2015).

## Supplementary Material

Supplementary Information

## Figures and Tables

**Figure 1:**
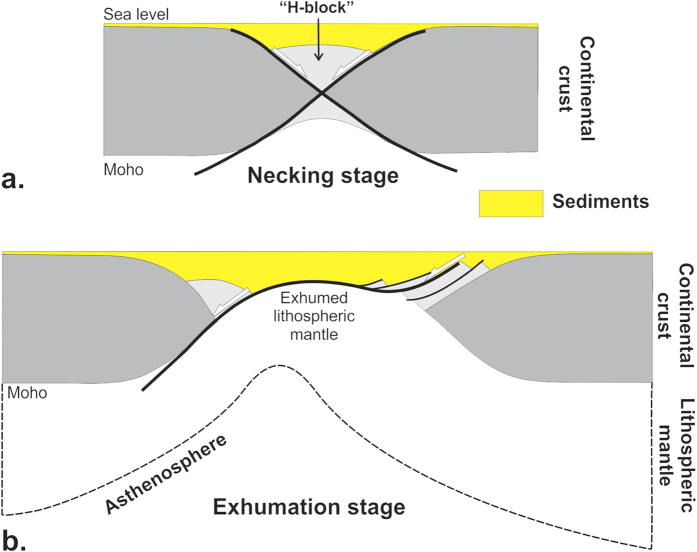
Necking (**a**) and mantle exhumation (**b**) stages at non-volcanic passive margins^13^,^14^. A tectonized continental block (H-Block) is isolated during the necking stage, bounded by conjugate master faults dipping “oceanward”. It is further dissected at both conjugate margins during a subsequent stage when bulk deformation becomes simple shear and lithospheric mantle is exhumed and serpentinized. All deformation is coeval with subsidence and sedimentation. From refs 13 and 14, modified (author: L.G., using CorelDraw11).

**Figure 2:**
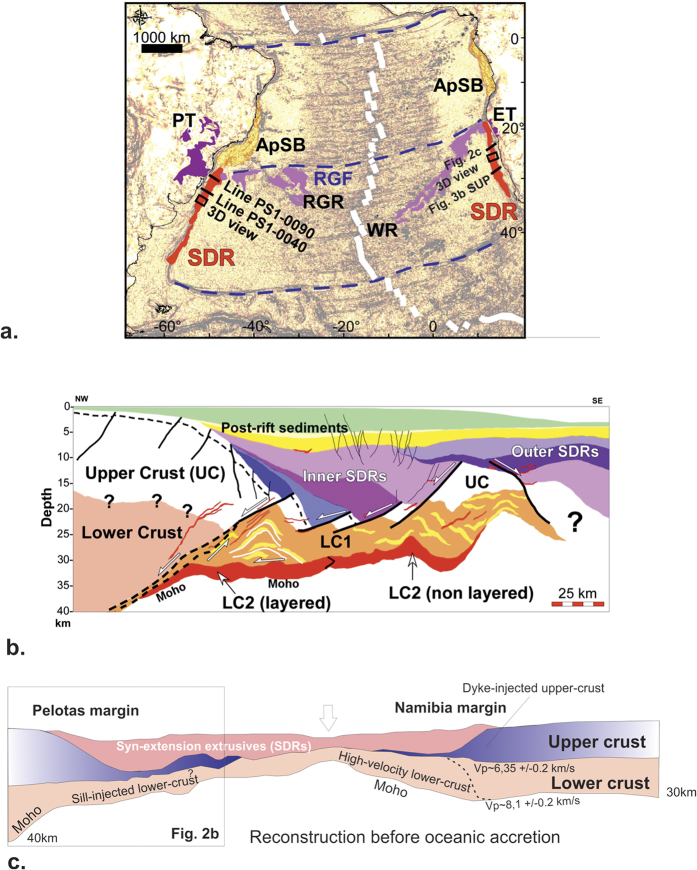
(**a**) Map of the first horizontal derivative of the Bouguer gravity field in the South Atlantic and location of the referred seismic profiles. PT, ET: Parana and Etendeka traps, respectively. WR, RGR: Walvis and Rio Grande Rises, respectively. APsB: Aptian Salt Basin. RGF not FTF: Rio Grande Transform. Author, P.W. using a software created by TOTAL. (**b**) Interpretation of PelotasSPAN line PS1-0090 (ION Geophysical). For original lines PS1-0040 and PS1-0090 along with interpretations, see Additional Data. Authors, P.W. and L.G., using CorelDraw11. (**c**) Crustal-scale profile of conjugate Pelotas and Namibia VPMs, during break-up. Figure is to scale. The arrow indicates the location of the earliest ocean-floor accretion. The Namibian profile^22^ is located in (**a**). Crustal structure and seismic velocities of Namibian margin are found in ref. 22. Authors L.G. and P.W, using CorelDraw11.

**Figure 4:**
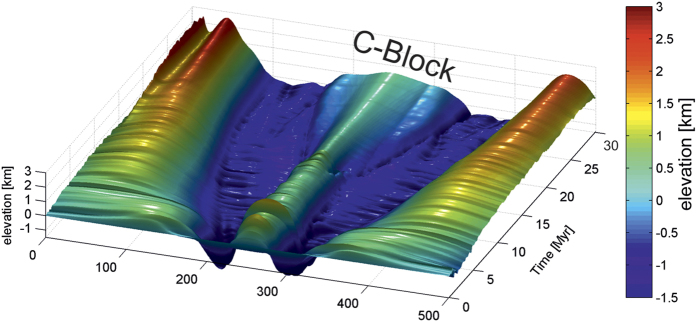
Evolution with time of basement elevation (see Methods). Note, with time, the deepening and widening of the SDR-related flexure, the flexural shoulder uplift and the long-term buoyancy of the C-Block (see Figs 3 and 5). Author: E.B. Image created from modelling results using Adobe Photoshop CS6.

**Figure 5:**
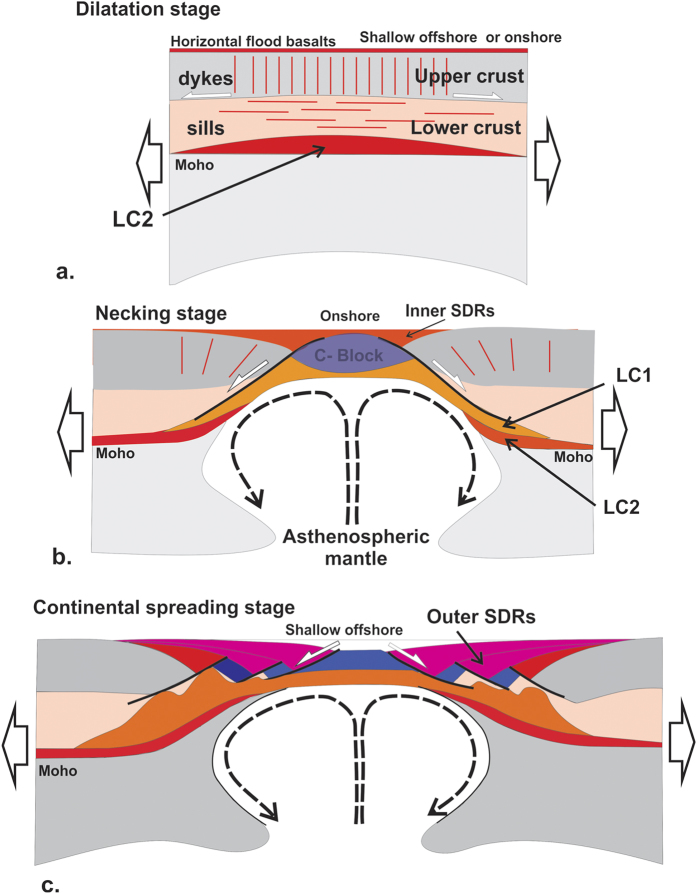
Proposed model of conjugate VPM formation. Note that asymmetry in the width and/or crustal thickness of conjugate VPMs often exists^20^. (**a**) Initial stage associated with minor tectonic extension but with intense dilatation of the crust by mafic magmas (sills and dyke swarms in the lower and upper crust, respectively). Flat-lying basaltic traps (typically, ~2 km thick^6^,^7^,^9^) are extruded at this time. (**b**) Extreme crustal thinning and stretching during the necking stage with individualization of inner SDRs and a central continental block (C-Block, see also Fig. 4), to be compared with the SPMs-related H-Block (Fig. 1a). (**c**) Continental spreading through fragmentation of the C-block owing to bulk pure-shear deformation with formation of the outer SDRs. Author: L.G., using CorelDraw11.
