# Supplementary material for: Volcanic passive margins: another way to break up continents
Source: Sci Rep. 2015 Oct 7;5:14828. doi: 10.1038/srep14828 (PMC4595843; doi:10.1038/srep14828)
Supplement: Supplementary Information [file srep14828-s1.doc]

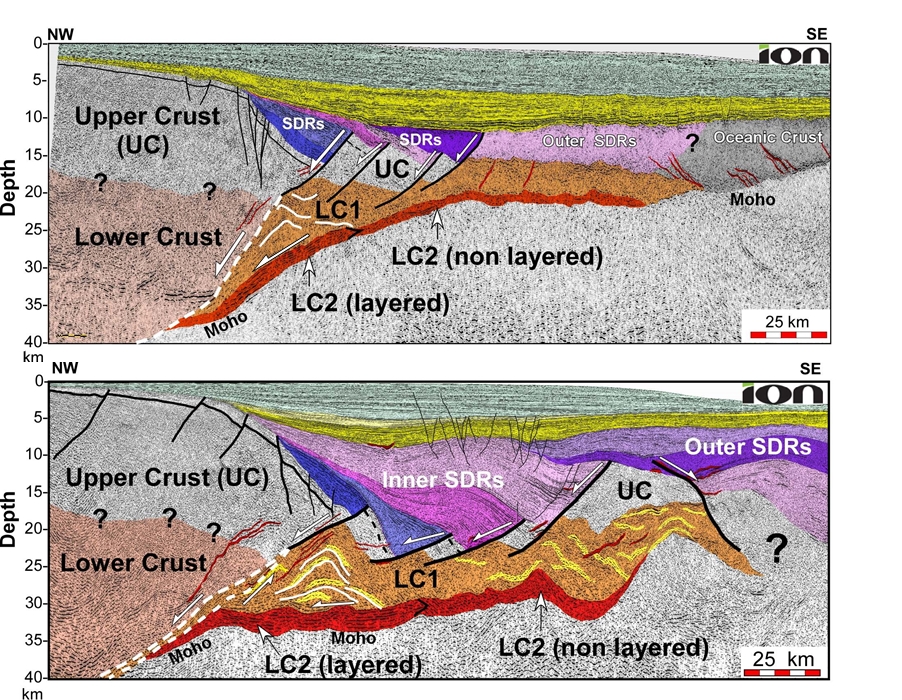


Figure 1 Extended Data


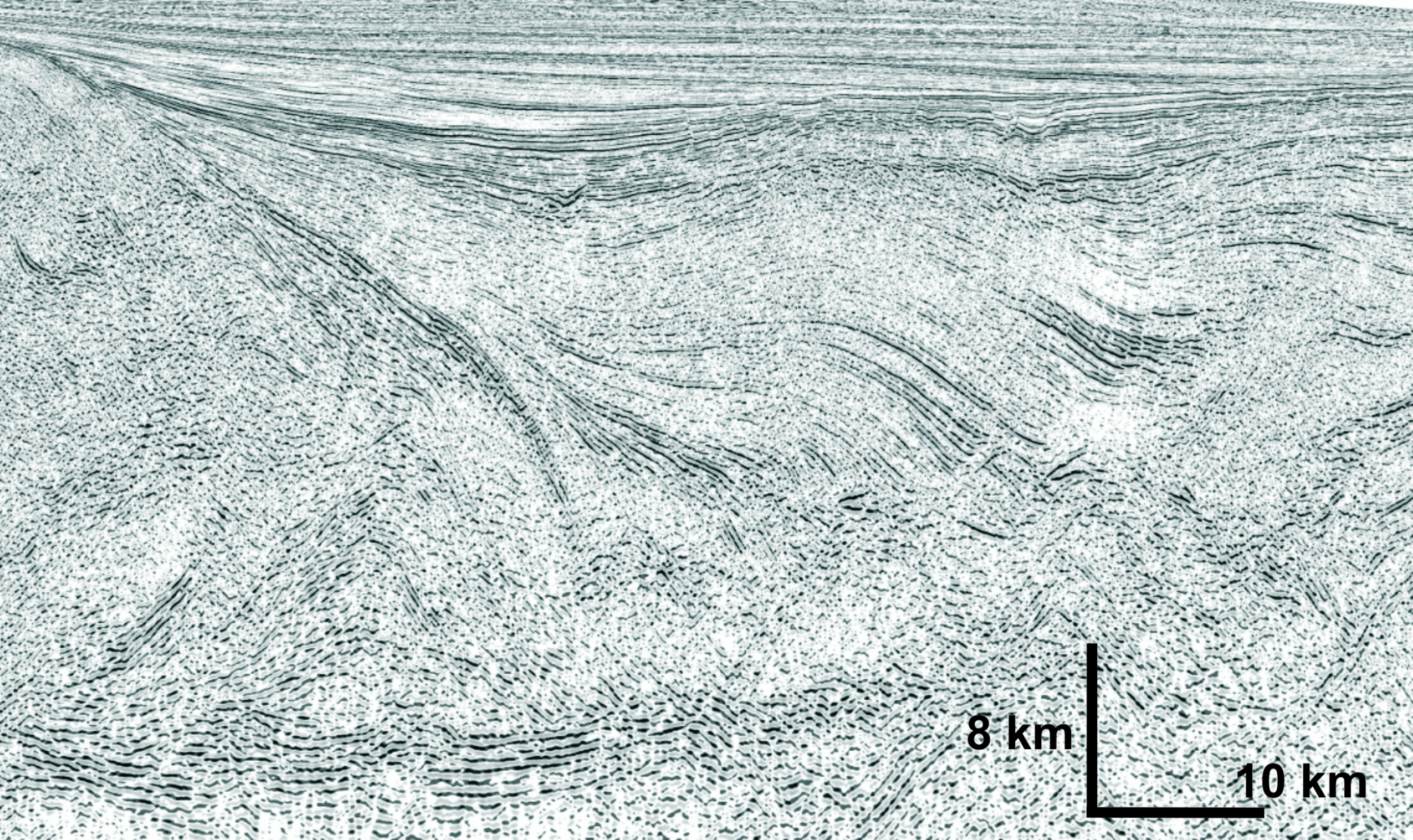


Figure 2 Extended Data


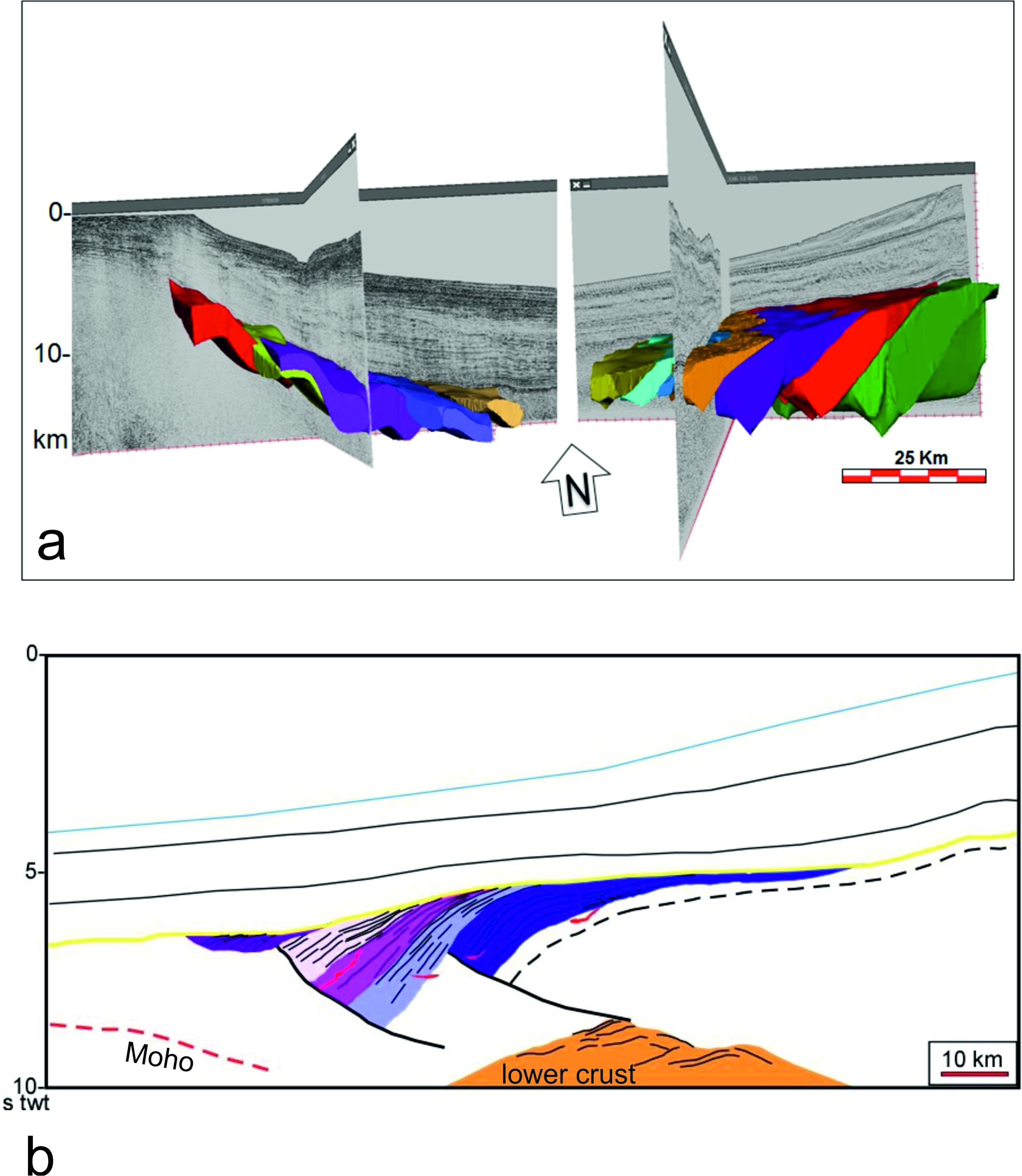


Figure 3 Extended Data


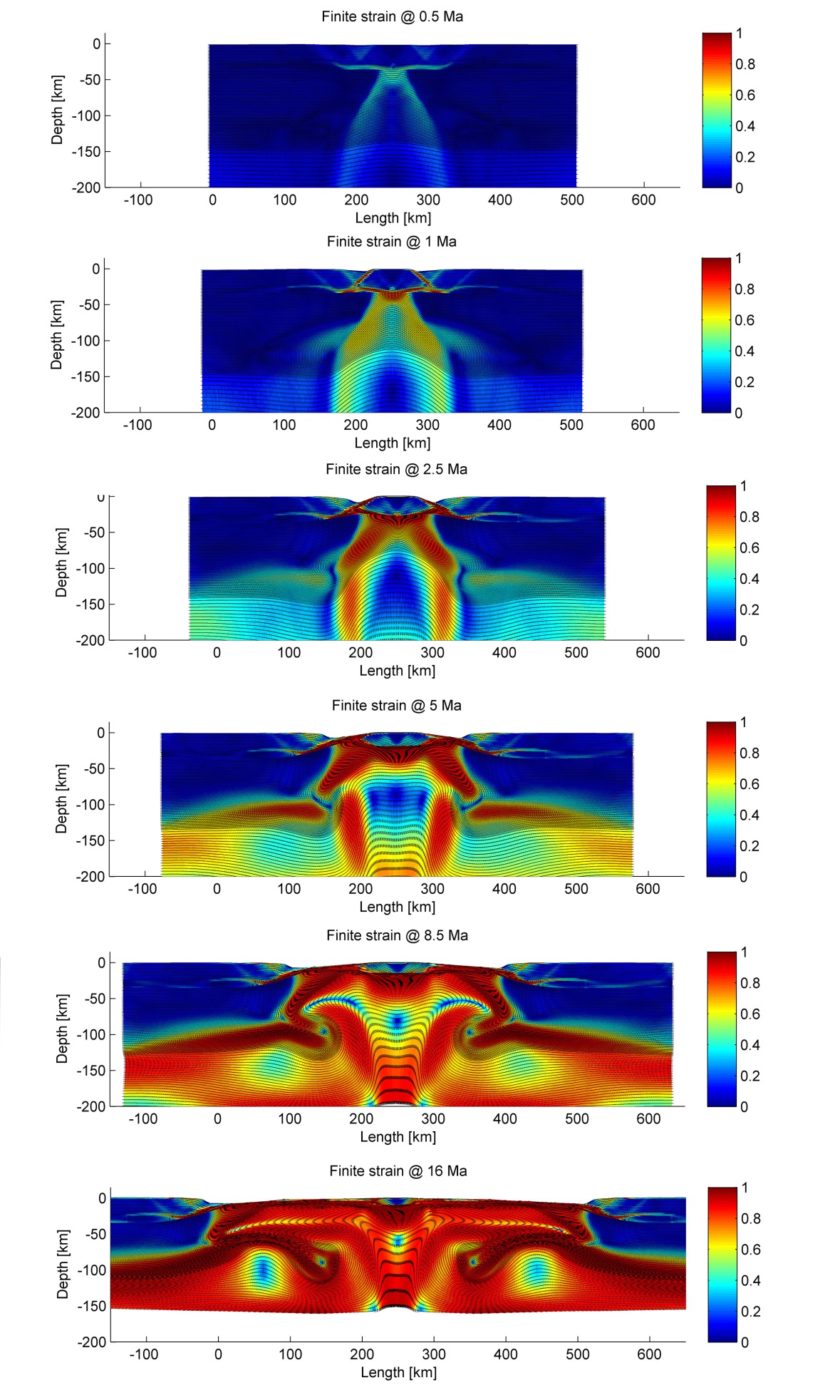


Figure 4. Extended Data

| **Extended Data Table 1. Thermomechanical coefficients and boundary conditions used in our models^50^.** | | |
| --- | --- | --- |
| Thermal | Surface temperature | 0°C |
|  | Temperature at the bottom of the thermal lithosphere | 1330°C |
|  | Thermal conductivity of crust | 2.5 Wm^-1^ °C |
|  | Thermal conductivity of mantle | 3.5 Wm^-1^ °C |
|  | Thermal diffusivity of mantle | 10^-6^ m^2^s^-1^ °C |
|  | Radiogenic heat production at surface | 1×10^-9^ W kg^-1^ |
|  | Radiogenic heat production decay depth constant | 10 km |
|  | Thermo-tectonic age of the lithosphere | 1000 Ma |
|  | Surface heat flow | 40 mW m^-2^ |
|  | Mantle heat flow | 15 mW m^-2^ |
| Mechanical | Density for all materials | ρ = *f* (P,T) calculated using Perple_X^43^ (kg m^-3^) |
|  | Lamé elastic constant λ, G (here, λ =G) | 30 GPa |
|  | Byerlee’s law – Friction angle | 30° |
|  | Byerlee’s law – Cohesion | 20 MPa |
|  | Plasticity, Peierls flow (τ_0_, *,* Q)^44^ | 9100 MPa, 1.3×10^-12^s^-1^, 498 kJ mol-1 |

| **Extended Data Table 2. Ductile creep parameters used in this study.** | | | | | |
| --- | --- | --- | --- | --- | --- |
|  | Composition | A | n | Q | Ref. |
|  |  | [MPa^-n^s^-1^] |  | [KJ.mol^-1^] |  |
| Upper  crust | dry quartzite | 6.8×10^-6^ | 3 | 156 | 45 |
|  | wet quartzite | 1.1×10^-4^ | 4 | 223 | 54 |
|  |  |  |  |  |  |
| Lower  crust | dry Maryland diabase | 8 ± 4 | 4.7 ± 0.6 | 485 ± 30 | 54 |
|  | dry diabase | 6.3×10^-2^ | 3.05 | 276 | 55 |
|  |  |  |  |  |  |
| Mantle | dry olivine | 1×10^4^ | 3 | 520 | 56 |
|  | wet olivine | 417 | 4.48 | 498 | 57 |

**Tables References**

54. Mackwell, S.J., Zimmerman, M.E. & Kohlstedt, D.L. High-temperature déformation of dry diabase with applications to tectonics on Venus. J. Geophys. Res., 103, 975–984, doi: 10.1029/97JB02671 (1998).

55. Carter N. L. & Tsenn M. C. Flow properties of continental lithosphere. Tectonophysics, 136, 27–63 (1987).

56. Wilks K. R. & Carter N. L. Rheology of some continental lower crust. Tectonophysics, 182, 55–77 (1990).

57. Chopra P. N. & Paterson M.S. The role of water in the deformation of dunite. J. Geophys. Res., 89 (B9) 7861–7876 (1984).

**Figure captions**

**Figure 1**. Upper box: Interpretation of ION Geophysical PelotasSPAN Line PS1-0040, and lower box: interpretation of ION Geophysical PelotasSPAN Line PS1-0090. UC: Upper Crust, LC1 and LC2: middle and deep lower crust. Profiles are located in Fig. 2a of the manuscript. Authors: P.W and L.G., using CorelDraw11 and imaging software created by TOTAL.

**Figure 2.** Enlarged extract from ION Geophysical PelotasSPAN Line PS1-0090 (see Figure 1 above) illustrating some distinctive features of VPMs such as large syn-volcanic continentward dipping detachment fault and the underlying deformed lower crust**.** Author: L.G., using CorelDraw11 and imaging software created by TOTAL.

**Figure 3.**

a: 3D view of inner and outer SDR wedges from South-Atlantic conjugate margins (Pelotas Basin-Namibia). Location in Figure 2a of the manuscript. The different SDR bodies are built up from the interpretation of an orthogonal set of 2D seismic lines. Although a relative similarity and symmetry can be observed at the large scale, a detailed study shows large variations in thicknesses both transversally and longitudinally to the margin axis within the SDR wedges. b: Example of a seismic profile with SDRs and CDFs located to the south of the (a) 3D view (location in Figure 2a of the manuscript). Images created by P.W.using a software created by TOTAL.

**Figure 4.** Snapshots of modeled finite deformation at steps from 0.5 Ma to 16 Ma. Images created from modelling results using Adobe Photoshop CS6.
